# A Mechanistic Digital Twin of uPAR-Driven Prostate Cancer Invasion Integrating ODE Signalling and Agent-Based Modelling

**DOI:** 10.3390/ph19030395

**Published:** 2026-02-28

**Authors:** Radosław Dzik, Joanna Chwał, Ewaryst J. Tkacz, Sudeep Roy, Agata Kabała-Dzik

**Affiliations:** 1 Department of Clinical Engineering, Academy of Silesia, ul. Rolna 43, 40-555 Katowice, Poland; joanna.chwal@akademiaslaska.pl (J.C.); ewaryst.tkacz@wst.pl (E.J.T.); 2 Faculty of Electrical Engineering and Communication, Brno University of Technology, Technická 3058/10, 616 00 Brno-Královo Pole, Czech Republic; roy@vut.cz; 3 Department of Pathology, Faculty of Pharmaceutical Sciences in Sosnowiec, Medical University of Silesia in Katowice, Ostrogórska 30, 41-200 Sosnowiec, Poland; adzik@sum.edu.pl

**Keywords:** uPAR, prostate cancer, agent based modelling, ODE, digital twin

## Abstract

**Background**: Aberrant signalling through the urokinase-type plasminogen activator receptor (uPAR) is a key driver of tumour invasion and progression in prostate cancer, yet linking molecular-level perturbations to emergent spatial invasion phenotypes remains challenging. **Methods**: In this study, we developed a multiscale in silico framework combining molecular docking, mechanistic ordinary differential equation (ODE) modelling, and agent-based modelling (ABM) to investigate uPAR-driven invasion dynamics. **Results**: Molecular docking and MM-GBSA analyses were used to prioritise caffeic acid phenethyl ester (CAPE) as a candidate uPA/uPAR modulator, while uPAR inhibition was implemented mechanistically at the signalling level within the ODE model rather than through direct energetic parametrisation. Steady-state signalling outputs were mapped to effective proliferation and motility rates, which served as inputs to a spatial ABM of tumour invasion. The integrated simulations showed that uPAR inhibition results in statistically significant reductions in spatial invasion and tumour growth compared with baseline conditions, whereas enhanced uPA signalling produced only modest, non-significant trends. **Conclusions**: These findings demonstrate how subtle intracellular signalling perturbations can translate into pronounced population-level invasion phenotypes when embedded in a spatial context. Overall, the proposed digital-twin framework provides a coherent and extensible approach for connecting molecular prioritisation with quantitative predictions of tumour invasion behaviour in prostate cancer.

## 1. Introduction

Epidemiological data show that prostate cancer is a high-incidence malignancy, typically listed as the second most common cancer affecting men worldwide. Prostate cancer often follows a slow, insidious course, and early-stage disease may produce few or no symptoms, resulting in many cases being identified only once the tumour has advanced [1]. Prostate cancer represents a leading male cancer diagnosis worldwide because it develops from non-aggressive to aggressive forms with mechanisms that move through surrounding tissues and ECM and spread to distant locations. A key challenge in advanced prostate cancer is the ability of tumour cells to invade surrounding tissue, traverse the extracellular matrix (ECM), migrate, intravasate, and ultimately colonise distant sites. Among the molecular systems that facilitate these processes, the urokinase-type plasminogen activator receptor (uPAR) axis stands out as a central regulator of both pericellular proteolysis and intracellular signalling. The urokinase-type plasminogen activator system operates at the interface of extracellular proteolysis and intracellular signal transduction. The cell-membrane receptor uPAR functions as a glycosylphosphatidyl-inositol (GPI)-anchored protein which binds urokinase-type plasminogen activator (uPA) to perform proteolytic functions at cell surfaces and to transmit signals that govern cell adhesion, migration, survival, and proliferation [2]. The expression levels of uPAR proteins rise as cancer advances and metastasizes, leading to poorer outcomes for patients across multiple cancer types [3]. In prostate cancer specifically, the uPA/uPAR system has been shown to be up-regulated in malignant tissue compared to benign or normal prostate tissue, and its overexpression is associated with increased invasiveness and metastatic potential. Mechanistically, uPAR does not simply act via proteolysis of the ECM, but via a complex network of interactions as follows: uPAR engages integrins, G-protein-coupled receptors, and growth factor receptors, thereby triggering signalling cascades such as FAK/Src, PI3K/AKT, MAPK/ERK, and NF−κB, which in turn promote proliferation, survival, migration, and epithelial-mesenchymal transition (EMT) [4]. Despite the substantial body of work describing uPAR expression and function in vitro and in vivo, there is still a gap remaining in our ability to quantitatively link molecular-level uPAR signalling dynamics to higher-order phenomena such as tumour cell population behaviour, invasive front formation, and response to therapeutic modulation. In other words, while many experimental studies illustrate that uPAR is a “driver” of invasion, few frameworks provide a mechanistic, quantitative, predictive model that spans from molecular signalling to cell population dynamics.

Before conducting the simulations, molecular docking was performed for the evaluated phytochemicals (flavonoids/polyphehols)—CAPE (caffeic acid phenethyl ester), quercetin, apigenin, naringenin, and chrysin—to assess their binding affinity to uPAR and to select the most promising candidate.

The research establishes a complete computer-based system which combines (i) an ordinary differential equation (ODE) model of uPAR-initiated signalling in prostate cancer cells and (ii) an agent-based model (ABM) of tumour cell population dynamics and invasion in heterogeneous microenvironmental conditions. The ODE model triggers uPAR → integrin/FAK → PI3K/AKT, ERK, and NF−κB pathways which result in efficient phenotypic changes of proliferation and motility. The ABM model uses these rates as input parameters to simulate prostate cancer cell population behaviour in space and time while considering different uPAR expression levels and environmental factors and treatment effects. The model studies uPAR modulation by inhibitor methods to understand their impact on tumour invasion which may suggest the optimal intervention scenarios in the future. Such a multiscale in silico model holds promise for enhancing our mechanistic understanding of prostate cancer invasion driven by uPAR, and for informing future experimental design and therapeutic strategies. Ultimately, it may contribute to the development of a predictive “digital twin” of uPAR-mediated invasion in prostate cancer and the promising usage of natural compounds.

The term digital twin is used here to denote a mechanistic, dynamically simulated computational counterpart of a biological system that preserves causal structure across multiple biological scales. In this work, the digital twin integrates molecular-level binding information, intracellular signalling dynamics described by ordinary differential equations, and emergent invasion behaviour captured through agent-based modelling. Unlike purely statistical or phenomenological models, this framework maintains mechanistic continuity from receptor-level perturbation to population-level phenotype. While the present implementation does not yet incorporate patient-specific calibration, it fulfils the core conceptual criteria of a digital twin as a continuously interrogatable, scenario-responsive in silico representation of a defined biological process. As such, it represents a mechanistic prototype digital twin of uPAR-driven prostate cancer invasion, designed to support hypothesis testing, sensitivity analysis, and future personalisation.

## 2. Results

Induced Fit Docking (Table 1) of phytochemical ligands to uPAR (PDB ID: 2FD6) revealed notable differences once receptor flexibility was considered. Although apigenin and naringenin showed more favourable rigid-receptor Glide scores, CAPE achieved the most favourable composite IFD score, indicating enhanced stabilisation following ligand-induced conformational adjustment of the binding site. Quercetin displayed intermediate binding behaviour, while chrysin yielded the least favourable IFD score.

**Figure 1:**
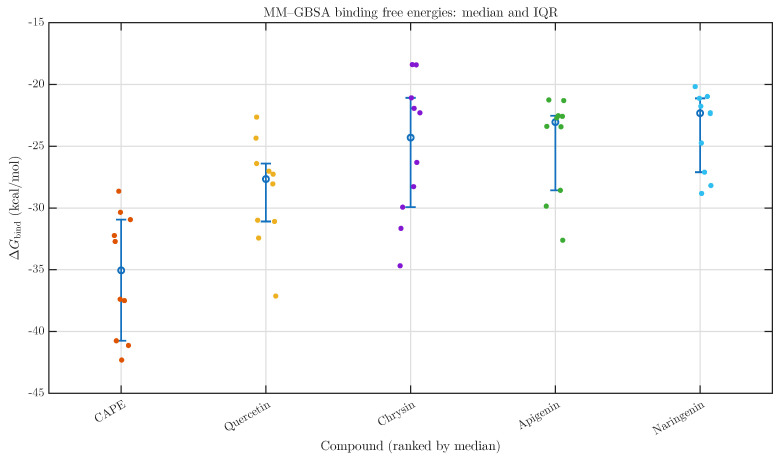
MM-GBSA binding free energies of phytochemical-uPAR complexes visualised as median and interquartile range. For each compound (*n* = 10), the central marker denotes the median MM-GBSA binding free energy ($\Delta G_{bind}$), while error bars represent the interquartile range (IQR; 25–75th percentiles), reflecting the dispersion of the central 50% of replica values. Individual points correspond to results from five independent simulation replicas.

The MM-GBSA calculations produced binding free energies which were studied for five phytochemicals through five separate simulations of each compound (raw values in Table A2). The evaluation of descriptive statistics showed that $\Delta G_{bind}$ values between ligands followed distinct patterns because their central values and distribution ranges were different. The analysis used median $\Delta G_{bind}$ values from compound ranking due to a small sample size and it showed moderate variation between replicate experiments. The study presents the mean ± SD values for additional information, although median $\Delta G_{bind}$ ± IQR values serve as the main ranking metric (Table 2). This ranking showed CAPE as the leading compound because it produced the most negative binding free energy values compared to all tested ligands. The binding free energy values of quercetin came in second place, followed by naringenin, chrysin, and apigenin, which had decreasingly negative median $\Delta G_{bind}$ values (Figure 1). The ordering pattern followed the typical values which the simulations produced while CAPE maintained stable binding properties throughout different simulation tests.

Post hoc Dunn comparisons with Benjamini–Hochberg correction were performed to quantify pairwise differences in MM-GBSA binding free energies between CAPE and the remaining phytochemicals (Table 3). CAPE exhibited significantly more favourable binding free energies than all four flavonoids. Adjusted *q*-values were $1.17\times 10^{-4}$ for naringenin, $8.81\times 10^{-4}$ for apigenin, $8.81\times 10^{-4}$ for chrysin, and $4.96\times 10^{-2}$ for quercetin. In each comparison, the negative Z-statistics (ranging from −1.96 to −4.18) indicate that CAPE consistently achieved more negative (i.e., stronger) $\Delta G_{\mathrm{bind}}$ values relative to the comparator compounds. The strongest statistical separation was observed for CAPE versus naringenin (Z=−4.18), followed by chrysin (Z=−3.49) and apigenin (Z=−3.41). Although the CAPE–quercetin comparison yielded the smallest effect magnitude (Z=−1.96), it remained statistically significant after multiple-testing correction (q=0.0496), indicating a modest but reliable advantage of CAPE over quercetin. The magnitude of separation was further quantified using median differences and non-parametric effect sizes. CAPE demonstrated the largest median shift relative to naringenin (ΔMedian =−12.73 kcal/mol; Cliff’s δ=−0.98), followed by apigenin (−12.01 kcal/mol; δ=−0.90) and chrysin (−10.75 kcal/mol; δ=−0.82). Although the CAPE–quercetin comparison yielded the smallest median difference (−7.40 kcal/mol), it remained statistically significant after correction and still exhibited a large effect size (δ=−0.70). According to standard non-parametric thresholds, all comparisons correspond to large effect magnitudes, indicating robust distributional separation rather than marginal statistical differences.

**Figure 2:**
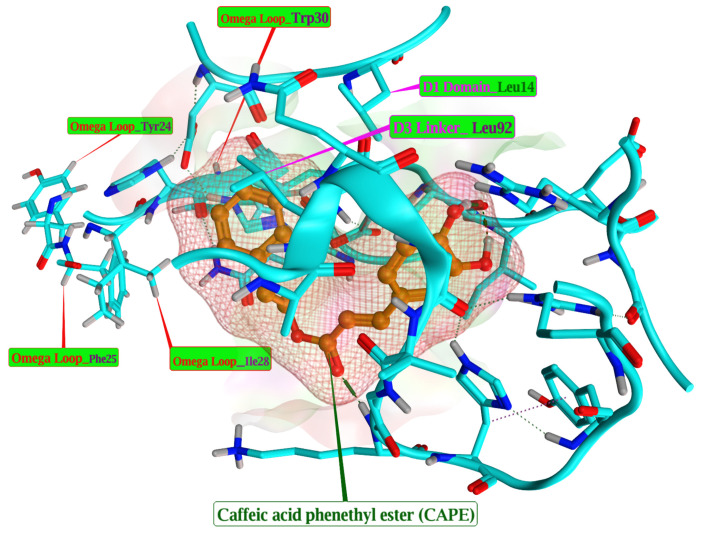
Interaction diagram of uPAR–CAPE complex. Docking and simulations were performed using Schrödinger Release 2025–1 and interactions were evaluated for hydrogen bonding, hydrophobic contacts, and ionic interactions.

On these bases (IFD and MM-GBSA), CAPE was prioritised as the most suitable uPAR-targeting candidate (Figure 2) for integration into the downstream multiscale digital-twin simulations. The interaction between uPAR and CAPE underscores the crucial role of essential hotspot residues such as Tyr24, Phe25, Ile28, and Trp30, which serve as the principal binding site for the Growth Factor Domain (GFD-Omega Loop) of uPA [5]. Together, these residues create a predominantly hydrophobic patch. Additionally, significant amino acids Leu14 (from Domain I) and Leu92 (from the Domain III-Linker region) [5] contribute to hydrophobic interactions with caffeic acid phenethyl ester (CAPE). Per-residue decomposition measures the individual contribution of each amino acid to the total binding free energy ($\Delta G_{bind}$) between a protein receptor and a ligand. These values highlight the critical hotspot residues vital for ligand recognition and binding affinity. A value of ΔG of < −2.0 kcal/mol indicates strong, favourable hotspot residues that are key drivers of binding (see Table A3 and full data in repository).

**Figure 3:**
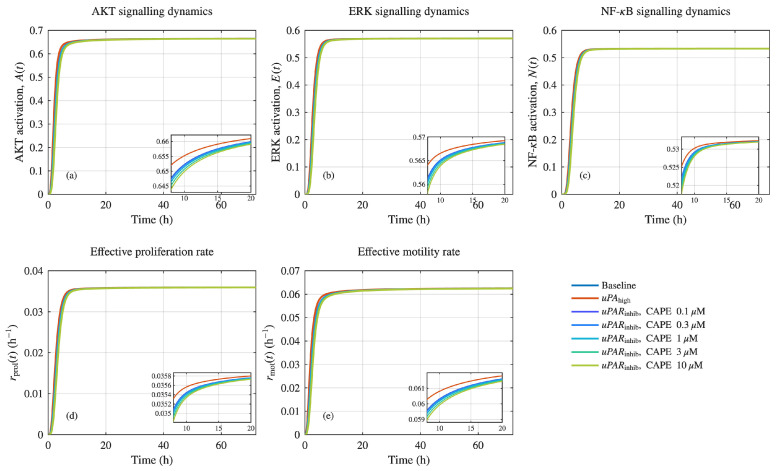
Intracellular uPAR signalling dynamics and phenotypic rate mapping. Time-dependent activation profiles downstream of uPAR are shown for baseline (blue), $uPA_{high}$ (orange), and $uPAR_{\mathrm{inhib}}$ conditions induced by CAPE (0.1–10 μM; colour gradient from purple/blue at low dose to yellow/green at the highest dose). Panels display (**a**) AKT, (**b**) ERK, and (**c**) NF-κB dynamics, followed by the derived effective phenotypic rates governing (**d**) proliferation $r_{\mathrm{prol}}\left(t\right)$ and (**e**) motility $r_{\mathrm{mot}}\left(t\right)$. Insets show magnified late-time dynamics (8–20 h), highlighting subtle but systematic differences in steady-state signalling and phenotypic rates across conditions. Time-averaged steady-state values (24–72 h) were baseline ${\bar{r}}_{\mathrm{prol}}=0.035894\;h^{-1}$ and ${\bar{r}}_{\mathrm{mot}}=0.062282\;h^{-1}$; ${\bar{r}}_{\mathrm{prol}}=0.035889\;h^{-1}$ and ${\bar{r}}_{\mathrm{mot}}=0.062253\;h^{-1}$.

The intracellular uPAR signalling model underwent numerical integration which showed that all downstream pathways became active quickly after the ligand bound to the system (Figure 3). The simulation produced identical signalling trajectory patterns which included baseline and $uPA_{high}$ and $uPAR_{inhib}$ conditions that showed immediate activity increases for AKT, ERK, and NF−κB before reaching stable levels between 8–12 h. The signalling trajectories followed identical patterns throughout all simulation conditions because the network maintained its stable operational behaviour. The systems generated identical brief answers through their operation, but they developed independent operational methods when operating continuously. The three signalling pathways demonstrated small increases in their plateau values when cells were exposed to $uPA_{high}$ conditions, yet uPAR inhibition resulted in a continuous decrease of pathway activation. These differences were subtle in the full time-course plots but became clearly visible upon magnification of the late-time window (8–20 h), as shown in the inset panels of Figure 3a–c. We used intracellular signalling state mapping to show that small variations between the two systems produced detectable changes which affected cellular reactions. The proliferation rate $r_{prol}\left(t\right)$—which depends mainly on AKT and NF−κB activity—established a lower equilibrium level when uPAR was blocked than when compared to the baseline and $uPA_{high}$ conditions (Figure 3d). The effective motility rate $r_{mot}\left(t\right)$—which depends on ERK and FAK/Src signalling pathways—showed decreased values in all experiments that used uPAR inhibitors (Figure 3e).

Time-averaged effective proliferation and motility rates (${\bar{r}}_{\mathrm{prol}}$, ${\bar{r}}_{\mathrm{mot}}$) derived from the ODE layer are reported in Table A4 for all simulated scenarios. Under baseline conditions, ${\bar{r}}_{\mathrm{prol}}=0.035894$ and ${\bar{r}}_{\mathrm{mot}}=0.062282$, while elevated uPA input produced only a marginal increase in both rates (${\bar{r}}_{\mathrm{prol}}=0.035909$, ${\bar{r}}_{\mathrm{mot}}=0.062362$). In contrast, occupancy-based uPAR inhibition introduced a concentration-dependent increase in the inhibition parameter η (0.0636–0.6364 across 0.1–10 μM CAPE), leading to progressive reductions in both effective rates. Although the absolute changes in ${\bar{r}}_{\mathrm{prol}}$ and ${\bar{r}}_{\mathrm{mot}}$ were modest, these fractional decreases became biologically consequential when propagated through the spatial ABM layer, consistent with the non-linear amplification observed in invasion metrics.

The agent-based model received its distinct phenotypic inputs from the small yet persistent absolute differences which appeared in steady-state rates.

**Figure A1:**
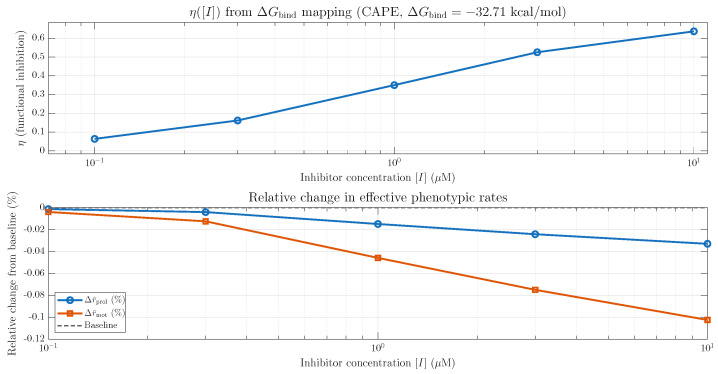
Agent-based model (ABM) invasion outcomes under different uPAR signalling scenarios. Occupancy-based functional inhibition (η) derived from MM-GBSA binding free energy and corresponding relative changes in ODE-derived effective proliferation and motility rates. Although steady-state rate reductions are modest (<1%), they scale monotonically with inhibitor concentration and provide the mechanistic input for multiscale amplification observed at the population level.

The occupancy-based mapping of docking-derived binding free energy into a functional inhibition parameter (η) produced a monotonic (Figure A1), concentration-dependent attenuation of uPA–uPAR association, with η increasing from 0.06 at 0.1 μM to 0.64 at 10 μM for CAPE. Although the resulting steady-state reductions in effective proliferation and motility rates were modest (<0.1% relative to baseline), these fractional changes provided the mechanistic input for multiscale amplification observed at the population level.

**Figure 4:**
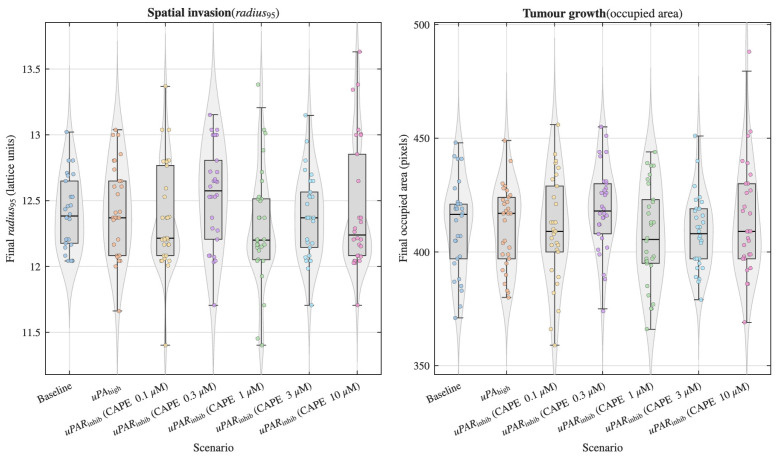
Multiscale propagation of uPAR inhibition into spatial tumour dynamics. The plots show the distribution of final invasion radius ($radius_{95}$) and occupied area across ABM simulations (n = 30 per scenario). Violin shapes represent kernel density estimates, boxes indicate median and interquartile range, and points denote individual simulation replicates coloured by scenario. Across increasing inhibition strength (η), only modest shifts in spatial metrics were observed, indicating attenuation of intracellular signalling suppression at the population level.

The agent-based simulations integrated the ODE-derived phenotypic rates to evaluate how intracellular signalling differences propagate to population-level tumour behaviour. Two complementary invasion endpoints were analysed as follows: spatial expansion quantified by the final invasion radius ($radius_{95}$) and tumour growth quantified by the final occupied area (Figure 4). Across stochastic replicates (n = 30 per scenario), graded uPAR inhibition produced only modest shifts in spatial invasion metrics relative to baseline. Although slight variations in median $radius_{95}$ and occupied area were observed across inhibition strengths, confidence intervals broadly overlapped and no statistically significant suppression of invasion emerged within the biologically plausible η range.

To further investigate whether intracellular uPAR inhibition propagates non-linearly to the population scale, we performed a threshold analysis linking inhibition strength (η) to spatial invasion dynamics. For each η level, we quantified the median shift in final invasion radius relative to baseline and estimated the 95% bootstrap confidence intervals. In addition, non-parametric effect sizes (Cliff’s δ) were calculated to assess the magnitude of deviation independently of distributional assumptions. To evaluate potential switch-like behaviour, a segmented step model was fitted to the median shifts, and an objective threshold criterion was applied based on confidence interval exclusion and effect size magnitude.

**Figure 5:**
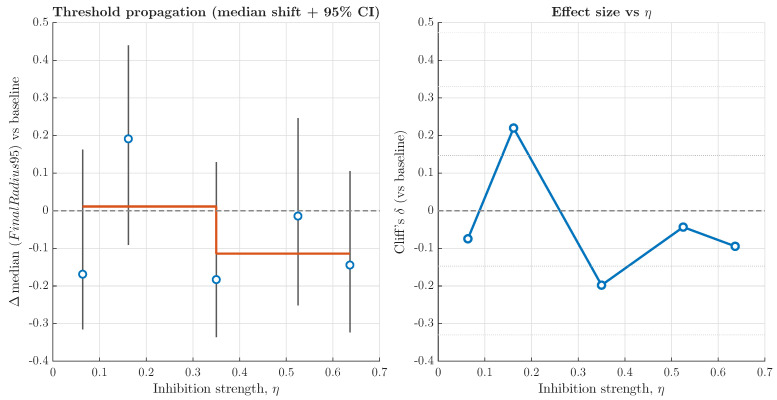
Threshold analysis of multiscale propagation from intracellular uPAR inhibition to spatial tumour invasion. (**left**): Median shift in final invasion radius (Δ median $radius_{95}$ vs. baseline (dashed line)) across increasing inhibition strength η, with bootstrapped 95% confidence intervals. The orange segmented line illustrates the best step-function approximation of a potential switch-like transition. (**right**): Corresponding non-parametric effect size (Cliff’s δ, blue line) relative to baseline (dashed). Across the explored η range, confidence intervals overlap zero and effect sizes remain within the small-to-moderate regime, indicating attenuated but non-critical propagation without a sharp threshold transition.

As shown in Figure 5, low-to-moderate inhibition strengths produced only minor fluctuations around baseline behaviour. However, beyond approximately η≈0.35, a consistent negative shift in invasion radius becomes visible, indicating measurable attenuation of spatial expansion. Although confidence intervals remain partially overlapping with zero, the trend toward sustained inhibition at higher η values suggests progressive multiscale propagation rather than a sharp bifurcation.

Non-parametric statistical analysis supported these observations. Kruskal–Wallis tests indicated a significant global effect of signalling condition on both spatial invasion and tumour area (Table 4). Post hoc Dunn tests with Benjamini–Hochberg correction identified uPAR inhibition as producing a statistically significant reduction in invasion relative to baseline for both $radius_{95}$ and occupied area ($q_{BH}<0.05$). In contrast, $uPA_{high}$ exhibited only a non-significant trend toward increased invasion compared with baseline, consistent with the modest elevation observed in ODE-derived phenotypic rates.

To assess sensitivity to spatial discretisation, we repeated ABM simulations across increasing lattice sizes (200 × 200, 300 × 300, 400 × 400; n = 30 per scenario). Median invasion radius and occupied area varied minimally across domains (typically <2% relative difference). Effect size analysis using Cliff’s δ showed predominantly negligible to small effects (|δ|<0.2 in most cases), with no monotonic dependence on lattice size. These findings indicate that tumour expansion remained boundary-free within the 72 h horizon and that invasion metrics are robust to lattice scaling under the present settings.

Taken together, these results demonstrate how small, steady-state differences in uPAR-mediated intracellular signalling can be amplified through phenotypic rate mapping and accumulated over time to generate distinct invasion outcomes at the tissue scale. Although signalling perturbations produced only subtle shifts in proliferation and motility rates, their integration within the agent-based framework led to statistically significant differences in emergent tumour spread. The integrated multiscale results are summarised quantitatively in Table 4, which links ODE steady-state outputs, ABM invasion metrics, and statistical significance in a single framework.

To assess whether lattice discretisation influenced invasion metrics, we performed a lattice-size sensitivity analysis by repeating the ABM simulations for L = 200, L = 300, and L = 400 while keeping all biological parameters unchanged. Only minor fluctuations were observed across lattice sizes, with effect sizes L = 400 relative to L = 200 remaining negligible throughout (Figure A3).

**Figure A2:**
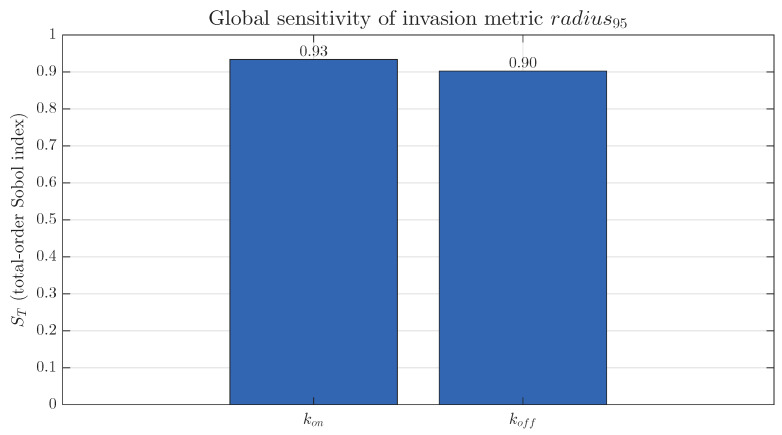
Total-order Sobol indices ($S_{T}$) for the invasion endpoint $radius_{95}$ demonstrating strong sensitivity to uPA–uPAR association ($k_{on}$) and dissociation ($k_{off}$) kinetics under biologically plausible parameter ranges (Saltelli *N* = 1024; five ABM replicates per sample).

**Figure A3:**
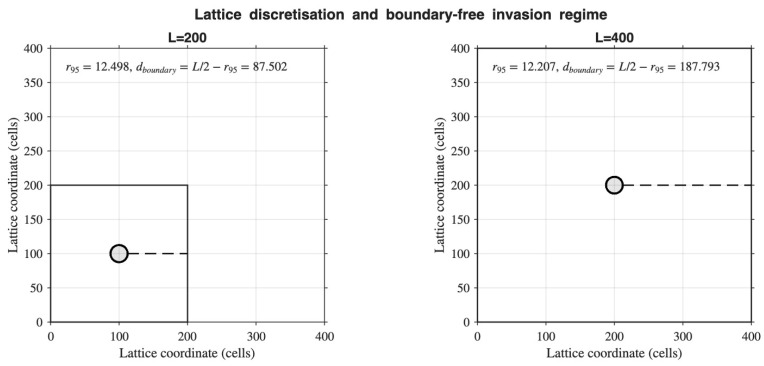
Lattice discretisation and boundary-free invasion regime. Schematic representation of the ABM domain for lattice sizes L = 200 and L = 400 showing a representative final tumour footprint (solid circle) and the corresponding $r_{95}$ invasion contour. In both cases, the boundary clearance $d_{\mathrm{boundary}}=L/2-r_{95}$ remains substantially larger than the invasion radius, indicating that the simulated tumour occupies only a small fraction of the computational domain.

Variance-based global sensitivity analysis was performed to quantify the influence of receptor binding kinetics on the primary invasion endpoint $radius_{95}$ (Figure A2). Total-order Sobol indices ($S_{T}$) revealed that both the association rate constant $k_{on}$ and the dissociation rate constant $k_{off}$ exert strong control over spatial tumour expansion, with $S_{T}$ values of 0.93 and 0.90, respectively. These high total-order indices indicate that invasion dynamics are predominantly governed by uPA–uPAR binding kinetics, including both direct effects and interaction contributions within the multiscale framework.

Together, these findings establish a mechanistic chain linking molecular-scale uPAR modulation to emergent invasion phenotypes, motivating a deeper interpretation of CAPE prioritisation within the multiscale digital-twin framework.

## 3. Discussion

The urokinase-type plasminogen activator receptor (uPAR) serves as a primary factor which enables tumours to invade and metastasize because it allows them to degrade extracellular matrix (ECM) and directs cell migration and regulates intracellular signalling pathways [6,7]. Research has shown that uPAR overexpression occurs in various cancer types which leads to more aggressive disease progression and worse patient results thus establishing its role as a cancer mechanism and treatment opportunity [8,9]. The cellular process of uPA-uPAR binding leads to FAK/Src and PI3K/AKT/ERK signalling pathway activation which regulates cell proliferation and movement patterns that define invasive cells.

In this study, we established a multiscale workflow integrating in silico molecular docking (MM-GBSA and IFD) for candidate selection with mechanistic ODE modelling of intracellular uPAR signalling and an agent-based model (ABM) of tumour invasion. Importantly, MM-GBSA binding free energies were used only to prioritise CAPE as a plausible uPAR inhibitor and were not directly parametrised into the ODE signalling model; instead, we encoded uPAR inhibition as a mechanistic perturbation in the signalling network, preserving interpretability and avoiding arbitrary mapping of energetics onto kinetic rates.

We made a purposeful decision to prevent MM-GBSA binding free energy calculations from becoming kinetic parameters because of the chosen modelling approach. MM/GBSA and MM/PBSA are widely used as binding free energy estimators and docking re-scorers, serving effectively as filters and hypothesis-generation tools in virtual screening workflows rather than predictors of kinetic behaviour [10,11]. The study of molecular-scale equilibrium thermodynamics uses binding free energies to understand uPAR-driven signalling dynamics which stem from non-equilibrium processes that involve receptor clustering and co-receptor recruitment and pathway cross-talk [6]. Directly mapping $\Delta G_{bind}$ values onto rate constants would therefore introduce unjustified assumptions and risk overfitting [12]. Instead, molecular docking serves here as a filtering and hypothesis-generation layer, identifying compounds likely to perturb uPAR function, while the ODE model captures the functional consequence of such perturbations through mechanistic attenuation of uPA–uPAR signalling flux. This separation preserves biological interpretability and ensures that downstream phenotypic effects arise from network dynamics rather than arbitrary energetic scaling.

Steady-state analysis of the ODE model demonstrated that alterations in uPAR activity produced modest but persistent shifts in key signalling intermediates when viewed in the late time window. Such differences, while subtle in the full time courses, were revealed more clearly through the inset panels, emphasising the importance of steady-state resolution in multiscale dynamics. Translating these signalling shifts into effective proliferation and motility rates enabled consistent coupling between the signalling and ABM components of the framework, as has been emphasised in other hybrid and agent-based approaches to cancer modelling [13,14].

The ABM results revealed that uPAR inhibition leads to statistically significant reductions in both spatial invasion and occupied area relative to baseline, consistent with non-parametric statistical analyses. In contrast, enhanced uPA signalling produced only non-significant trends toward increased invasion, suggesting that moderate perturbations in signalling strength may be modulated by spatial constraints and cell-cell interactions inherent to the ABM. These findings are congruent with computational studies where spatial structure and microenvironmental interactions shape emergent tumour behaviours beyond what intracellular signalling alone might predict [14,15].

To contextualise the multiscale modelling findings, it is important to interpret our molecular docking results in light of existing biological evidence. Caffeic acid phenethyl ester (CAPE), the top-ranked compound in our MM-GBSA and induced-fit docking analyses, shows promising activity against cancer-related signalling pathways. CAPE has been reported to suppress Akt and EGFR/FAK signalling, inhibit proliferation, and reduce tumour growth in prostate cancer cell models, suggesting a mechanistic foundation for its role as a uPAR-targeting agent [16,17,18]. These molecular activities are consistent with our docking results indicating stable binding poses and favourable interaction energies. The ODE-ABM digital-twin framework uses molecular evidence to convert CAPE-associated inhibition into functional effects which affect signalling pathways and tissue structures. The uPAR inhibition scenario from in silico simulations which used CAPE prioritisation results in decreased AKT and ERK and NF−κB signalling levels that produce lower effective cell proliferation and movement rates. The agent-based model shows statistically significant decreases in tumour invasion and occupied area when we used these rates in model inputs instead of default values. The combination of molecular docking with dynamic multiscale modelling demonstrates that CAPE and other agents which disrupt uPA/uPAR signalling pathways can effectively stop prostate tumour progression through specific quantitative measurements which surpass traditional molecular binding assessments.

From a molecular perspective, the prioritisation of CAPE cannot be interpreted solely in terms of favourable docking scores, but rather in terms of where and how CAPE interacts with uPAR. Both induced-fit docking and MM-GBSA residue decomposition indicate that CAPE preferentially engages hydrophobic hotspot residues within the uPA growth factor domain (GFD) recognition region, including Tyr24, Phe25, Ile28, and Trp30. These residues are known to mediate high-affinity uPA–uPAR binding and to stabilise receptor conformations that favour integrin coupling and downstream signal propagation [4,5]. By occupying this interface, CAPE is therefore positioned to interfere not only with pericellular proteolysis, but also with uPAR-dependent co-receptor signalling assemblies involving integrins and growth-factor receptors [3,4]. This spatial and mechanistic specificity distinguishes CAPE from ligands that bind more peripherally or rely predominantly on polar interactions, and it provides a plausible structural basis for its downstream functional impact.

The strength of the proposed digital-twin framework lies in its ability to amplify subtle molecular perturbations into measurable tissue-scale phenotypes. At the ODE level, CAPE-associated uPAR inhibition produces only modest reductions in the steady-state activation of AKT, ERK, FAK/Src, and NF−κB. However, when these altered signalling states are mapped onto effective proliferation and motility rates, even small fractional decreases become biologically consequential, consistent with prior multiscale signalling-to-phenotype modelling approaches [13,15]. Within the agent-based model, these reduced rates are applied iteratively over many stochastic time steps, leading to cumulative effects on cell dispersal, invasive front roughness, and spatial expansion, as widely reported in agent-based models of tumour invasion [14,19].

Although uPA-uPAR signalling has been widely implicated in tumour invasion and metastasis through its role in extracellular matrix degradation and cell migration, with elevated uPAR expression often correlating with increased malignancy in multiple cancer types [6], our multiscale simulations showed only modest, non-significant propagation of inhibition into spatial invasion metrics. The threshold analysis did not reveal an abrupt transition in invasion behaviour with an increase in inhibition strength; instead, shifts in median invasion emerged gradually and effect sizes remained small, consistent with multiscale modelling frameworks in which intracellular perturbations are attenuated across biological scales rather than producing sharp macroscopic transitions [20].

This behaviour illustrates a central principle of multiscale cancer dynamics as follows: non-linear amplification across biological scales allows weak intracellular perturbations to generate strong emergent outcomes when embedded in spatial and collective cellular contexts.

In the present study, MM-GBSA binding free energy calculations were deliberately used as a comparative prioritisation and structural hypothesis-generation tool, rather than as fully converged, absolute thermodynamic estimates. MM-GBSA approaches combine molecular mechanics energies with continuum solvation models and are widely employed to re-score and refine docking poses in virtual screening workflows, offering intermediate accuracy between simple scoring functions and rigorous free energy perturbation methods, albeit with known approximations and limitations in entropy and solvent treatments [10,11]. Consequently, the resulting $\Delta G_{bind}$ values should be interpreted as effective affinity proxies that aid structural interpretation and ligand prioritisation, not as experimentally validated binding constants. Importantly, our prioritisation of CAPE was supported by structural convergence criteria beyond ΔG magnitude. Specifically, CAPE consistently engages hydrophobic amino acids within the urokinase growth factor-like domain (GFD) that have been identified as hot-spot residues in mediating high-affinity uPA–uPAR interactions, including Tyr24, Phe25, Ile28, and Trp30 [21,22]. These residues form a predominantly hydrophobic patch in the Ω-loop of uPA’s GFD that interfaces with complementary regions of uPAR and have been implicated in stabilising the high-affinity receptor–ligand complex. Engagement of these conserved interaction motifs by CAPE–in addition to its induced-fit stability across multiple replicas–provides mechanistic plausibility for its functional prioritisation, irrespective of small differences in ΔG. We acknowledge that increased sampling depth and alternate free energy methodologies could further refine these estimates. However, given the known approximations of MM-GBSA and the absence of biochemical constants for many studied ligands, the current approach strikes a balance between computational feasibility and mechanistic insight.

To quantitatively link predicted molecular affinity with functional inhibition in the ODE model, we adopted an occupancy-based mapping from estimated binding free energies to fractional receptor occupancy. Classical receptor occupancy theory establishes that the biological effect of a ligand is proportional to the fraction of receptors it occupies, which at equilibrium can be described in terms of the equilibrium dissociation constant ($K_{d}$) and ligand concentration using standard ligand–receptor models (fractional occupancy = $\left[L\right]/(K_{d}+\left[L\right])$) in pharmacodynamic modelling frameworks [23]. Models that explicitly separate affinity ($K_{d}$) from downstream responses, have been used to interpret how variations in equilibrium binding influence functional outcomes, especially in quantitative receptor and pharmacodynamic models [24]. By converting MM-GBSA derived $\Delta G_{bind}$ into an effective $K_{d}$ and computing fractional occupancy under assumed inhibitor concentrations, we provide a physically grounded, widely accepted mechanistic connection between molecular energetics and the modulation of uPA–uPAR signalling within the digital-twin framework.

To assess the robustness of invasion dynamics in our multiscale framework, we performed a global sensitivity analysis using variance-based Sobol total-order indices, which—unlike one-at-a-time local methods—quantifies parameter influence over wide biologically plausible ranges and accounts for non-linearities and interactions [25]. Our results demonstrate that invasion phenotypes are strongly influenced by receptor association and dissociation kinetics, underscoring the regulatory importance of binding dynamics and the value of comprehensive sensitivity assessment in mechanistic cancer invasion modelling. Our findings resonate with global sensitivity studies in systems biology that highlight how variance-based methods such as Sobol decomposition systematically identify influential parameters in multiscale models, and suggest that biologically relevant variation in uPA–uPAR binding kinetics can translate into substantive differences in invasion phenotypes through their integrated effect on signalling and motility dynamics [3,25].

The negligible lattice-size dependence confirms that invasion dynamics are governed by intrinsic proliferation–motility balance rather than domain confinement. Because the simulated tumours occupied only a small fraction of the computational domain and lattice choice was informed by standard practices to avoid boundary effects [26,27], boundary effects did not contribute to endpoint variability. This strengthens confidence that the modest multiscale propagation observed in our framework reflects biological attenuation rather than numerical artefacts of lattice resolution or domain size, consistent with prior multiscale agent-based analyses in the literature [28]. To determine whether lattice discretisation affected the invasion readouts, we conducted a lattice-size sensitivity analysis, while maintaining identical biological and model parameters across all runs. Median $r_{95}$ and final area values remained highly consistent across lattice sizes, with only minor fluctuations and negligible effect sizes relative to L = 200. The boundary clearance $d_{\mathrm{boundary}}=L/2-r_{95}$ remained substantially larger than the invasion radius in all cases, confirming that tumour expansion occurred in a boundary-free regime and that invasion endpoints were not driven by domain constraints.

### Limitations

Our ODE–ABM digital-twin framework is organised into two mechanistically distinct but sequentially coupled layers as follows: a receptor-level signalling module and a spatial invasion module. The ODE system provides a reduced yet mechanistically interpretable representation of uPA–uPAR complex formation and downstream signalling flux, translating receptor perturbation into effective proliferation and motility rates. These rates are subsequently propagated into the agent-based model (ABM), where invasion emerges from stochastic cell–cell and cell–space interactions. This separation of scales prevents molecular parameter overfitting while preserving a direct, physically grounded link between compound-level perturbation and tissue-scale invasion phenotypes.

Model parameters were calibrated using literature-derived kinetic ranges and experimentally constrained signalling behaviour, while uncertain parameters were confined to biologically plausible bounds rather than fitted to optimise phenotypic outputs. The present implementation should therefore be regarded as a proof-of-concept mechanistic framework rather than a fully calibrated predictive simulator. In this context, the term “digital twin” refers specifically to a structured computational representation of the uPA–uPAR signalling process and its multiscale consequences, rather than to a patient-specific or clinically individualised therapeutic model.

The ODE component intentionally adopts a parsimonious structure focused on the minimal receptor–ligand interaction required to drive invasion-relevant signalling outputs. Although uPAR biology can involve receptor clustering, co-receptor recruitment, and feedback regulation within MAPK or NF−κB pathways, these mechanisms were not explicitly incorporated due to limited quantitative calibration data and concerns regarding parameter identifiability. Similarly, the current ABM does not yet include extracellular matrix remodelling or immune-cell interactions, which represent biologically relevant extensions for future development. The model should therefore be interpreted as a mechanistically grounded but reduced representation of uPAR-driven invasion dynamics.

Despite these structural simplifications, the integration of docking-based compound prioritisation with a mechanistic multiscale framework enables quantitative assessment of how molecular perturbations propagate across scales. By linking binding affinity–derived inhibition to signalling attenuation and ultimately to spatial invasion metrics, the framework demonstrates how receptor-level modulation can generate measurable differences in tissue-level behaviour, providing an extensible computational prototype of a signalling-process digital twin for invasion modelling.

## 4. Materials and Methods

### 4.1. Rationale and Differences Between the Evaluated Phytochemicals

The selected phytochemicals differ in both chemical structure and physicochemical properties, which are expected to influence their interaction with uPAR. CAPE is a phenolic ester with high lipophilicity, facilitating strong hydrophobic interactions within the uPAR binding pocket. Quercetin and apigenin are flavones/flavonols with multiple hydroxyl groups, enabling extensive hydrogen bonding but potentially reducing membrane permeability. Naringenin is a glycosylated flavonoid with higher molecular weight and polarity, which may limit deep penetration into the binding cavity despite favourable surface interactions. Chrysin, characterised by fewer hydroxyl groups, exhibits lower polarity and a simpler interaction profile, often resulting in weaker binding affinity. These structural differences justify their comparative docking evaluation, as they provide insight into how molecular size, polarity, and functional group composition affect uPAR binding and enable rational selection of the compound most suitable for downstream dynamic modelling and digital-twin simulations.

### 4.2. Biological Assumptions

In the present study we model a prostate cancer cell phenotype representative of advanced, highly invasive, androgen-independent disease. To that end, we adopt the PC-3 cell line as the biological referent for our in silico framework.

The following assumptions underlie that choice:

1. Aggressive metastatic derivation
  The PC-3 cell line was originally established from a bone metastasis of a human prostate adenocarcinoma (grade IV) and is thus representative of a late-stage, metastatic context [29]. Moreover, PC-3 cells have been widely characterised as having higher metastatic and invasive potential compared to less aggressive prostate cancer cell lines [30].
2. Androgen independence and loss of AR signalling
  PC-3 cells are classically regarded as androgen receptor (AR)-negative and largely unresponsive to androgen stimulation, making them an appropriate in vitro model of castration-resistant prostate cancer [31]. The androgen-independent status simplifies model assumptions by eliminating the need to explicitly model androgen AR-axis feedback in our in silico study.
3. High uPA/uPAR system activity and invasive phenotype
  Comparative analyses of prostate cancer cell lines demonstrate that PC-3 cells express significantly higher levels of the urokinase-type plasminogen activator (uPA) and its receptor uPAR, and show correspondingly elevated in vitro invasiveness (e.g., Matrigel invasion) compared to lines such as LNCaP [32]. Specifically, it has been shown that the malignant phenotype of prostate tumour cells correlates with the expression of uPA and uPAR, for example, in PC-3 and DU145 lines versus less invasive lines [33]. These traits align with our modelling focus on uPAR-driven invasion and proteolytic/motility mechanisms.
4. Relevance to multiscale modelling of invasion
  Because our modelling framework integrates (i) intracellular signalling downstream of uPAR (such as FAK/Src, PI3K/AKT, MAPK/ERK) and (ii) population-level invasion dynamics (cell motility, ECM degradation, and spatial expansion), the PC-3 phenotype offers both the intracellular flux (high signalling throughput) and the spatial/invasive behaviour needed to justify agent-based modelling of an aggressive tumour front.
5. Simplification-friendly phenotype for in silico modelling
  The androgen-independent and highly invasive phenotype of PC-3 cells provides a consistent baseline for in silico parametrisation. Specifically, by modelling a “PC-3-like” cell—defined here as high uPAR expression, high motility/proteolytic capability, AR-independent context—we reduce complexity in the model by not incorporating androgen—AR regulatory loops. Instead, the model can focus solely on uPAR-mediated mechanisms of invasion, which is central to our study aim.

As such, in our model, each agent in the population layer is assumed to represent a “PC-3-like” cell, characterised by high uPAR expression, elevated motility/invasion potential, and androgen-independent survival. The ODE signalling network is parametrised to reflect intracellular dynamics consistent with PC-3 cell behaviour (e.g., elevated uPA/uPAR activity and strong downstream activation of motility/invasion pathways). The agent-based model uses proliferation and motility rates derived from these intracellular parameters under different conditions (e.g., basal, uPA stimulation, and uPAR inhibition) to simulate the spatial–temporal invasive behaviour of the population.

### 4.3. Molecular Docking of Phytochemicals Targeting uPAR

Prior to dynamic simulations, an in silico molecular docking study was conducted utilising Schrödinger Suite 2025.1 (Schrödinger Release 2025-1: Maestro, Schrödinger LLC, New York, NY, US) to evaluate the binding potential of selected phytochemicals belonging to the flavonoid (quercetin, apigenin, naringenin, and chrysin) and phenolic acid derivatives, namely, CAPE (caffeic acid phenethyl ester), against the urokinase plasminogen activator receptor (uPAR). The three-dimensional structure of uPAR was obtained from the Protein Data Bank (PDB Id: 2FD6), and substrate structures were retrieved from publicly available chemical database PubChem [34]. Before proceeding with the docking, we thoroughly investigated all binding pockets, as well as the potential conformations and orientations of the substrates within the protein’s active sites. Flexible substrate docking was performed using Glide-XP (Extra precision), incorporating the OPLS3 force field [35,36]. To account for receptor flexibility and ligand-induced conformational changes within the uPAR binding site “Induced Fit docking” [37] was employed, generating 20 poses for each run. Additionally, redocking was performed on structures within 30.0 kcal/mol of the optimal pose, among the top 20 structures, to validate the docking results and ensure their reliability based on known uPA-uPAR interaction regions. The outcomes of the docking simulations were quantified using a scoring function known as GlideScore (GScore) [38]. Binding affinity scores and interaction profiles (hydrogen bonding, hydrophobic contacts, and π−π interactions) were analysed to rank compounds and to select the most promising uPAR-binding candidate for subsequent systems-level simulations.

Further, Molecular dynamics (MD) simulations were performed on protein–substrate docked complexes utilising Desmond software, version 4.7, which implements the OPLS3e force field (Schrödinger LLC, New York, NY, USA, 2020) [39,40]. The solvent, membrane, and periodic boundary conditions were tailored to meet the specific requirements. An SPC solvent model was integrated within an orthorhombic box containing the protein–substrate complex. Additionally, the system was neutralised by introducing appropriate counterions and maintaining a salt concentration of 0.15 M [41]. To prevent steric interactions, the distance between the protein–substrate complex and the box wall was carefully kept at 10 Å. The minimization stages facilitate the configuration and submission of jobs that stabilize the system into a local energy minimum. We adopted a hybrid strategy that integrates the steepest descent with the limited-memory Broyden-Fletcher-Goldfarb-Shanno (LBFGS) algorithms, achieving a gradient cut-off [42] of 25 kcal/mol over a period of 100 ps. The dynamics were analysed using a multi-time step RESPA integrator algorithm [43]. Throughout the simulations, we maintained a temperature of 300 K with Nose-Hoover thermostats while ensuring stable pressure utilising the Martyna-Tobias-Klein barostat method. We also defined both the short- and long-range Coulombic interactions. The NPT (number of particles, pressure, and temperature) ensemble was employed for 100 ns on the equilibrated system. Following this, relaxation took place under the constant NVT (number of particles, volume, and temperature) ensemble for 1 ns to generate simulation data for subsequent analysis [44,45]. For each compound, five independent simulation replicas were analysed to account for conformational variability and to assess the robustness of binding. The results were examined by using the simulation interaction diagram (SID) and trajectory plot module provided by Desmond.

To validate the findings from the Induced Fit docking, the binding free energies of the protein–substrate complexes were calculated using Prime-MM/GBSA (Molecular Mechanics/Generalized Born Surface Area) (Prime, Schrödinger, LLC, New York, NY, USA, 2020). The total binding free energy ($\Delta G_{bind}$) was computed as the sum of molecular mechanics energy terms (electrostatic, van der Waals, covalent contributions), solvation free energy, and lipophilic interactions. In addition to total $\Delta G_{bind}$, individual energy components were analysed to provide mechanistic insight into ligand–receptor interactions. We prioritised total MM-GBSA [11,46] binding free energy ($\Delta G_{bind}$) because it represents the net thermodynamic stability of the ligand-uPAR complex, whereas individual energy components are interdependent and primarily informative for mechanistic interpretation rather than compound ranking.

For quantitative comparison between compounds, $\Delta G_{bind}$ values were summarised as medians ± IQR across replicas. Because of the small sample size (*n* = 10 per compound) and the absence of assumptions regarding normality, non-parametric statistical analysis was performed.

Post hoc pairwise comparisons of MM-GBSA binding free energies were conducted using Dunn’s [47] test with Benjamini–Hochberg [48] correction for multiple comparisons. In addition to reporting adjusted *q*-values, effect sizes were quantified using median differences (ΔMedian) and Cliff’s δ, a non-parametric measure of stochastic dominance ranging from −1 to 1. Effect magnitude was interpreted according to established thresholds as follows: |δ|<0.147 (negligible), 0.147≤|δ|<0.33 (small), 0.33≤|δ|<0.474 (medium), and |δ|≥0.474 (large) [49].

### 4.4. Occupancy-Based Mapping of Binding Free Energy to Functional Inhibition

To provide a quantitative yet interpretable bridge between molecular docking results and the mechanistic ODE signalling model, we introduced an occupancy-based mapping that links estimated binding free energies to effective inhibition of uPA–uPAR complex formation.

MM-GBSA binding free energies ($\Delta G_{bind}$) were converted to effective dissociation constants ($K_{d}$) using the following thermodynamic relationship:

(1) $$K_{d}=\exp\left(\frac{\Delta G_{bind}}{RT}\right),$$

where $R=1.987\times 10^{-3}$$\mathrm{kcal}\cdot {\mathrm{mol}}^{-1}\cdot K^{-1}$ is the gas constant and T=300 K represents physiological temperature. We note that MM-GBSA estimates do not yield fully converged absolute thermodynamic quantities; therefore, the resulting $K_{d}$ values are interpreted as effective affinity proxies rather than experimentally validated dissociation constants.

Assuming reversible binding, fractional receptor occupancy (θ) at a given inhibitor concentration [*I*] was computed using a Langmuir-type isotherm as follows:

(2) $$\theta =\frac{\left[I\right]}{\left[I\right]+K_{d}}.$$

To account for potential partial antagonism, membrane accessibility constraints, or downstream signal buffering, occupancy was scaled by a maximal functional inhibition parameter ($\eta_{\max}$):

(3) $$\eta =\eta_{max}\cdot \theta ,$$

where $0\leq \eta_{max}\leq 1$.

Under this formulation, η corresponds to the fraction of effective uPA–uPAR association flux reduced by inhibitor binding. For example, η=0.7 reflects an approximate 70% reduction in receptor-mediated signalling flux at the specified concentration. Importantly, η does not imply complete pathway shutdown unless receptor occupancy approaches saturation. This formulation ensures biological plausibility while preserving separation between thermodynamic affinity estimation and dynamic kinetic modelling.

Because MM-GBSA absolute ΔG values may overestimate affinity, η was capped (as above) and a small residual association flux was retained to avoid artefactual full pathway shutdown while preserving relative inhibition trends.

The resulting compound-specific inhibition term η was implemented in the ODE model by attenuating the effective uPA–uPAR association rate as follows:

(4) $$k_{on}^{eff}=\left(1-\eta \right)k_{on}.$$

Inhibitor concentration [*I*] was treated as a scenario parameter rather than a calibrated experimental input. To avoid overinterpretation of absolute $K_{d}$ values derived from MM-GBSA, simulations were performed across a biologically plausible concentration range (0.1–10 μM), and invasion metrics were reported as concentration-dependent response curves.

This mapping provides a physically grounded yet assumption-transparent connection between molecular affinity estimates and network-level inhibition, while preserving separation between thermodynamic approximation and dynamic signalling kinetics.

### 4.5. Model Overview and Workflow

**Figure 6:**
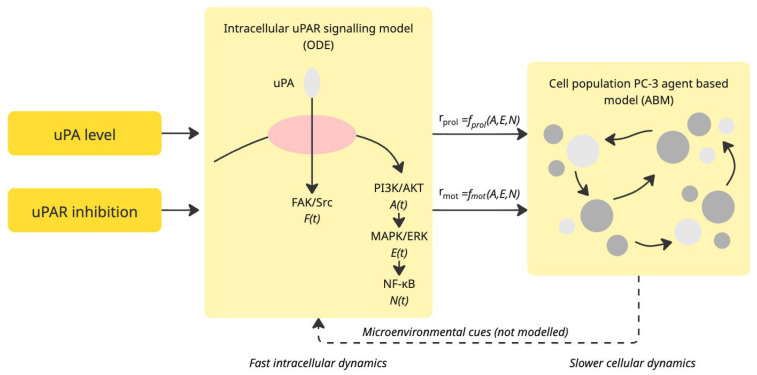
Multiscale in silico framework linking uPAR signalling dynamics to prostate cancer cell invasion. The model integrates intracellular uPAR-mediated signalling with population-level tumour behaviour. (**left**): External inputs include the extracellular uPA level and the degree of uPAR inhibition. Centre: The intracellular signalling module (ODE model) represents activation of the uPA-uPAR complex and downstream pathways, including FAK/Src, AKT, ERK, and NF−κB, which collectively determine effective phenotypic rates of proliferation $r_{prol}$ and motility $r_{mot}$. (**right**): These rates serve as inputs to an agent-based model (ABM) representing a population of PC-3-like tumour cells that undergo probabilistic migration, proliferation, quiescence, apoptosis, and interaction with their microenvironment. The integrated framework enables simulation of how perturbations of uPAR signalling propagate from molecular dynamics to emergent invasive tumour behaviour.

We developed a multiscale in silico framework that integrates intracellular uPAR-mediated signalling dynamics with population-level tumour cell invasion. The model consists of two interconnected components: (i) an ordinary differential equation (ODE) system describing the activation of key signalling pathways downstream of the uPA-uPAR axis (a system involving the urokinase-type plasminogen activator (uPA) and its receptor (uPAR)) in a PC-3-like prostate cancer cell, and (ii) an agent-based model (ABM) representing the spatial–temporal behaviour of a heterogeneous cell population undergoing proliferation, migration, and invasion. The overall workflow is illustrated conceptually in Figure 6. At the intracellular scale, the ODE model simulates the receptor–ligand interactions and downstream phosphorylation cascades associated with active uPAR, including FAK/Src, PI3K/AKT, MAPK/ERK, and NF−κB signalling modules. These biochemical time courses are reduced to two effective phenotypic descriptors as follows: an effective proliferation rate $r_{prol}$ and an effective motility rate $r_{mot}$. These rates represent the dominant functional outputs of uPAR-driven signalling and serve as the interface between molecular signalling and cell-level behaviour.

At the population scale, we implement an agent-based representation in which each cell is modelled as an autonomous agent occupying a location in a two-dimensional spatial domain. Each agent inherits its phenotypic parameters $r_{prol}$ and $r_{mot}$ from the ODE model, with optional heterogeneity introduced through distributions of uPAR expression or signalling responsiveness. Agents undergo probabilistic migration, proliferation, and, where applicable, quiescence or apoptosis, subject to local spatial constraints and microenvironmental cues. The coupling between the two scales is achieved through the following scenario-based mapping strategy: for each experimental condition (e.g., baseline, elevated uPA, partial, or complete uPAR inhibition), the intracellular signalling model is solved independently to obtain the corresponding phenotypic rates. These rates are then used to initialise and drive the ABM simulations. This modular structure allows us to explore how perturbations of uPAR signalling propagate from molecular to tissue scale, enabling systematic in silico experiments on tumour invasion dynamics. Together, the ODE and ABM layers provide a mechanistic yet computationally tractable digital representation of uPAR-driven prostate cancer invasion. The workflow explicitly links biochemical regulation to emergent multicellular behaviour, supporting predictive simulations and hypothesis generation regarding the impact of uPAR modulation on tumour progression.

### 4.6. ODE-Network Structure and State Variables

The signalling scheme uses established interactions which occur in the uPA/uPAR axis. The uPA-uPAR complex forms through uPA binding to uPAR receptors on cell surfaces and this complex interacts with integrins and focal adhesion components to initiate the process. The activation of FAK/Src through phosphorylation begins the process which leads to PI3K/AKT and MAPK/ERK cascade activation and subsequent NF−κB activation. The signalling pathways operate as a single system which controls cell survival and proliferation and guides cytoskeletal changes and cellular movement.

The ODE system monitors continuous state variables which consist of ligand amounts and receptor availability and receptor–ligand complexes and signalling pathway activators. A representative set includes the following: [uPA], [uPAR], [uPA:uPAR], $\left[FAK^{*}\right]$, $\left[AKT^{*}\right]$, $\left[ERK^{*}\right]$, $\left[NF-\kappa \beta^{*}\right]$, where the asterisk denotes the activated form of each signalling protein.

Reactions follow mass-action or Michaelis–Menten-type kinetics depending on the biological mechanism being modelled. Receptor–ligand binding and dissociation, phosphorylation events, and signal propagation are represented using standard biochemical reaction terms. The general structure of each equation is as follows:

(5) $$\frac{dX_{i}}{dt}=f_{i}\left(X_{1},X_{2},\ldots ,X_{n};\theta \right)$$

where $X_{i}$ denotes the concentration of species i, and θ represents kinetic parameters such as binding rates, activation constants, or deactivation rates. Because uPAR signalling involves both rapid phosphorylation events and slower downstream transcriptional changes, we designed the system to be compatible with stiff ODE solvers. The model focuses on short- to intermediate-timescale dynamics (minutes to hours), appropriate for capturing the rapid biochemical shifts that define cellular motility and proliferative potential.

Parameter values were selected from literature ranges for uPA/uPAR kinetics and downstream signalling cascades in prostate cancer and other solid tumours. When precise quantitative data were not available, we constrained parameters using the following:

(i) Biologically plausible bounds.
(ii) Known qualitative relationships in PC-3 cells (e.g., strong AKT and ERK phosphorylation under high uPA stimulation).
(iii) Calibration against steady-state behaviours consistent with experimental reports.

Initial concentrations were set to reflect a high-uPAR, highly invasive baseline corresponding to PC-3 cells. uPAR inhibition conditions were implemented by reducing the effective concentration of the uPA-uPAR complex via a multiplicative scaling factor applied to the binding rate or effective receptor availability.

All ODE simulations were performed using a stiff solver with adaptive step size–ode15, ode45 (MATLAB R2025a (MathWorks, Natick, MA, USA) with tolerances chosen to ensure numerical stability across wide parameter ranges. Simulations were run over a normalised 24-h biochemical timescale.

To identify the signalling components most strongly influencing downstream phenotypes, we performed global sensitivity analysis (Sobol or Morris screening) on key kinetic parameters. Sensitivity results were used both to validate the robustness of the mapping from signalling to phenotype and to guide reduction of overly sensitive or redundant pathways.

For each simulated condition (baseline, uPA stimulation, and partial or full uPAR inhibition), we extracted the time-dependent activation profiles of $AKT^{*}$, $ERK^{*}$, $FAK^{*}$, and $NF-\kappa B^{*}$. These trajectories were reduced to the following two effective phenotypic descriptors:

- An effective proliferation rate, $r_{prol}$, primarily modulated by AKT and NF−κB signalling.
- An effective motility rate, $r_{mot}$, shaped mainly by FAK/Src and ERK activation.

These rates serve as inputs to the agent-based model and represent the mechanistic translation of intracellular signalling into cell-level behavioural parameters.

The full equation set of the ODE system and list of variables are provided in Appendix A.

### 4.7. Phenotypic Mapping Between Intracellular Signalling and Cell-Level Behaviour

The ODE model produces time-dependent activation levels of key signalling intermediates associated with uPAR-driven invasion. To integrate this signalling information into the agent-based model (ABM), we map these intracellular states onto the following two effective phenotypic rates:

- The proliferation rate $r_{\mathrm{prol}}$.
- The motility rate $r_{\mathrm{mot}}$.

These rates encode how strongly uPAR-mediated signalling promotes cell-cycle progression and cell migration, respectively. Below we provide explicit formulae.

#### 4.7.1. Phenotypic Proliferation Rate

Proliferation is primarily governed by AKT (pro-survival, pro-growth) and NF−κB (transcriptional activator supporting cell-cycle progression).

We define the following:

(6) $$r_{\mathrm{prol}}=f\left(A,N\right),$$

where A=A(t) is active AKT, and N=N(t) is active NF−κB.

A convenient, biologically meaningful form is a normalised Hill-type combination, as follows:

(7) $$r_{\mathrm{prol}}=r_{\mathrm{prol}}^{\max}\left[\alpha_{A}\frac{A}{A_{\mathrm{tot}}}+\alpha_{N}\frac{N}{N_{\mathrm{tot}}}\right]$$

with the following:

- $0\leq \alpha_{A},\alpha_{N}\leq 1$.
- $\alpha_{A}+\alpha_{N}=1$ (weights describing relative influence).
- $r_{\mathrm{prol}}^{\max}$ the maximal biologically feasible proliferation rate.

Here, the biological interpretation is as follows:

Both AKT and NF−κB promote proliferation. Their contributions are weighted and normalised to total protein amounts so the rate naturally scales between 0 and $r_{\max}$.

#### 4.7.2. Phenotypic Motility Rate

Motility and invasion are predominantly driven by FAK/Src (adhesion turnover, cytoskeletal dynamics) and ERK (migration and matrix remodelling).

We therefore define the following:

(8) $$r_{\mathrm{mot}}=g\left(F,E\right),$$

where F=F(t) is active FAK and E=E(t) is active ERK.

A biologically consistent functional form is as follows:

(9) $$r_{\mathrm{mot}}=r_{\mathrm{mot}}^{\max}\left[\beta_{F}\frac{F}{F_{\mathrm{tot}}}+\beta_{E}\frac{E}{E_{\mathrm{tot}}}\right],$$

with the following:

- $0\leq \beta_{F},\beta_{E}\leq 1$,
- $\beta_{F}+\beta_{E}=1$,
- $r_{\mathrm{mot}}^{\max}$ the maximal motility rate.

The biological interpretation is as follows: both ERK and FAK/Src promote migration; their relative influence is tunable and normalised.

### 4.8. ODE Signalling Module and Phenotypic Rate Mapping

Intracellular uPAR signalling was modelled using a mechanistic ordinary differential equation (ODE) system comprising ligand–receptor binding and internalisation dynamics coupled to downstream pathway activation states. The state vector was defined as $y\left(t\right)={\left[L,R,C,F,A,E,N\right]}^{top}$, representing free ligand *L*, free receptor *R*, ligand–receptor complex *C*, and the normalised activities of FAK/Src (*F*), AKT (*A*), ERK (*E*), and NF−κB (*N*). Three simulated signalling scenarios were considered as follows: baseline, $uPA_{high}$, and $uPAR_{inhib}$, implemented via scenario-specific uPA scaling and inhibition strength η (baseline: uPA=1.0, η=0; $uPA_{high}$: uPA=2.0, η=0; and $uPAR_{inhib}$: uPA=1.0, η=0.7). In Mode 1, uPA scaled ligand production (i.e., $k_{L,prod}$ was multiplied by the scenario uPA factor), while ligand concentration was not clamped to uPA (i.e., $clamp_{L\to uPA}=false$). Initial conditions were set to $y\left(0\right)={\left[0,1,0,0,0,0,0\right]}^{top}$. ODE simulations were performed in MATLAB using the stiff solver ode15s over a 72 h horizon (t∈[0, 72] h) with relative and absolute tolerances of $10^{-6}$ and $10^{-9}$, respectively. To obtain robust summary quantities for coupling to the agent-based model, time-averaged outputs were computed over the interval 24–72 h. The 24–72 h window was selected to exclude early activation transients while preserving a sufficiently long quasi-steady regime for stable rate estimation prior to ABM coupling. Phenotypic coupling was implemented by mapping signalling activities to effective proliferation and motility rates using bounded rate functions with maxima $r_{prol,max}=0.06\;h^{-1}$ and $r_{mot,max}=0.08\;h^{-1}$, with convex pathway weights $\alpha_{A}=\alpha_{N}=0.5$ for proliferation and $\beta_{F}=\beta_{E}=0.5$ for motility.

The weights linking signalling activity to effective proliferation and motility rates were set to equal values ($\alpha_{A}=\alpha_{N}=0.5$; $\beta_{F}=\beta_{E}=0.5$) to reflect a symmetry-preserving baseline assumption in the absence of quantitative data supporting differential pathway dominance. This choice avoids introduction of additional free parameters at the ODE–ABM interface and maintains mechanistic interpretability. The mapping is therefore intended as a reduced projection from signalling state to phenotypic rate rather than a calibrated pathway-specific contribution model.

### 4.9. Agent-Based Tumour Invasion Model (ABM)

The population-level behaviour of tumour cells was simulated using a two-dimensional agent-based model that represents individual cells as autonomous agents capable of probabilistic migration, proliferation, quiescence, and apoptosis. The ABM captures spatial competition, local crowding effects, and emergent collective invasion patterns that arise from uPAR-driven motility dynamics. Simulations were conducted on a discrete two-dimensional lattice of size $L_{x}\times L_{y}$, where each lattice site may be occupied by at most one cell agent. Boundary conditions were set to be reflective on all sides, preventing agents from leaving the domain. The spatial scale corresponds conceptually to a local region of tumour tissue or an in vitro invasion field. Each agent is characterised by the following attributes: spatial coordinates, a proliferation rate $r_{\mathrm{prol}}$, a motility rate $r_{\mathrm{mot}}$, and a binary viability state (alive, quiescent, or apoptotic). Heterogeneity in signalling and behaviour is introduced by sampling $r_{\mathrm{prol}}$ and $r_{\mathrm{mot}}$ from distributions associated with differing uPAR levels. At each ABM time step Δt, an agent attempts cell division with probability $P_{\mathrm{divide}}=r_{\mathrm{prol}}\Delta t$. Division is successful only if an adjacent lattice site is unoccupied; if no neighbouring space is available, the agent enters a temporary quiescent state. Cell migration follows a biased random walk influenced by $r_{\mathrm{mot}}$. At each time step, an agent attempts movement with probability $P_{\mathrm{move}}=r_{\mathrm{mot}}\Delta t$. When movement occurs, the agent selects one of its neighbouring lattice sites either uniformly or biased toward chemotactic/ECM gradients, depending on the scenario. Movement is allowed only if the target site is unoccupied. Agents undergo apoptosis with a small probability per time step $P_{\mathrm{death}}$, representing background turnover. Additionally, agents may enter quiescence if persistent crowding prevents division or if $r_{\mathrm{prol}}$ is sufficiently low under uPAR inhibition. All agents are updated asynchronously to limit artefacts arising from synchronous movement and division. Each simulation consists of $T_{\mathrm{ABM}}$ time steps, with population attributes (cell count, invasion distance, and density fields) sampled at predefined intervals.

### 4.10. Coupling Strategy Between ODE and ABM

To integrate intracellular signalling with cell-level behaviour, we use a scenario-based coupling approach. For each biochemical condition (e.g., baseline signalling, elevated uPA, and partial or full uPAR inhibition), the intracellular ODE model is solved independently to produce time series for A(t), N(t), F(t), E(t). These signals are transformed into instantaneous proliferation and motility rates via the phenotypic mapping functions described previously. To ensure numerical stability and biological realism, we compute the time-averaged effective rates. Specifically, for each condition, we evaluate the following:

(10) $${\bar{r}}_{\mathrm{prol}}=\frac{1}{t_{2}-t_{1}}\int_{t_{1}}^{t_{2}}r_{\mathrm{prol}}\left(t\right)dt,\;{\bar{r}}_{\mathrm{mot}}=\frac{1}{t_{2}-t_{1}}\int_{t_{1}}^{t_{2}}r_{\mathrm{mot}}\left(t\right)dt,$$

where $\left[t_{1},t_{2}\right]$ is a selected post-transient window (e.g., 2–8 h). Numerical integration is performed using the trapezoidal rule on the ODE solver time grid. The resulting pair (${\bar{r}}_{\mathrm{prol}},{\bar{r}}_{\mathrm{mot}}$) serves as the fixed behavioural parameters for all agents in the ABM under that condition. This approach ensures a clean mapping between molecular perturbation and emergent multicellular behaviour, while avoiding the need to integrate ODEs for every agent during ABM execution.

### 4.11. In Silico Experimental Scenarios

We simulated a series of biologically motivated scenarios as follows:

1. Baseline (PC-3-like) signalling
  ODE parameters correspond to the high-invasiveness phenotype typical of PC-3 cells.
2. Elevated uPA stimulation
  Increased ligand concentration enhances complex formation and downstream activation, yielding larger ${\bar{r}}_{\mathrm{prol}}$ and ${\bar{r}}_{\mathrm{mot}}$.
3. uPAR inhibition
  Partial or full blockade of uPAR is simulated by scaling the effective binding rate $k_{\mathrm{on}}$ or receptor availability. Multiple inhibition strengths (e.g., 25%, 50%, 75%, and 90%) were tested.
4. Heterogeneous population
  A mixed population with varying uPAR expression levels, leading to a distribution of behavioural parameters.
5. Therapeutic optimisation
  Systematic analysis of inhibitor strength versus invasion suppression, sweeping η∈[0,1].

All simulations were performed with MATLAB R2025a software (MathWorks, Natick, MA, USA).

### 4.12. Agent-Based Tumour Invasion Model and Simulation Protocol

A two-dimensional lattice-based agent-based model (ABM) was used to simulate emergent tumour growth and invasion driven by ODE-derived proliferation and motility rates. Simulations were performed on a 200 × 200 lattice ($N_{x}=N_{y}=200$) for 72 h using a fixed time step of Δt=0.5 h (144 steps total). Each signalling condition (baseline, $uPA_{high}$, $uPAR_{inhib}$) was simulated using n=30 independent stochastic replicates to account for intrinsic randomness, with a fixed master random seed (randomSeed=1) for reproducibility. Initial tumour seeding comprised 150 agents (initCells=150) placed as a compact disk of radius eight lattice units around the domain centre. At each time step, agent proliferation and movement probabilities were determined by the corresponding scenario-specific ODE-derived rates. Cell motility was implemented using a von Neumann neighbourhood ($moveNeighborhood=‘‘vonNeumann^{\prime \prime }$), while a small baseline death process was included with hazard $p_{death}=0.002$ $h^{-1}$. Simulation outputs were recorded at regular intervals (every 10 steps), including occupancy snapshots and summary invasion metrics computed at the final time point. Spatial invasion was quantified using the $radius_{95}$ endpoint (radius containing 95% of tumour agents), and tumour growth was quantified as the final occupied area (number of occupied lattice sites).

### 4.13. Global Sensitivity Analysis of Invasion Dynamics

To assess the robustness of the multiscale framework and quantify the influence of kinetic uncertainty on emergent invasion behaviour, we performed a variance-based global sensitivity analysis of the primary invasion endpoint, $radius_{95}$. Sensitivity was evaluated with respect to key uPA–uPAR binding kinetics, specifically the association rate constant ($k_{on}$) and dissociation rate constant ($k_{off}$), which govern receptor complex formation in the ODE layer.

Parameter uncertainty was represented by sampling each kinetic constant within biologically plausible bounds defined as multiplicative ranges (0.3×−3.0×) around baseline values. Sampling was conducted using the Saltelli extension of the Sobol quasi-random scheme, with a base sample size of N=1024, resulting in N(d+1) model evaluations for d=2 parameters. For each sampled parameter set, the ODE system was solved to steady state, and the resulting effective proliferation and motility rates (${\bar{r}}_{\mathrm{prol}}$, ${\bar{r}}_{\mathrm{mot}}$) were propagated to the agent-based model (ABM). Because the ABM is stochastic, five independent simulations were performed per parameter set using distinct random seeds, and the median final invasion radius ($radius_{95}$) was used as the sensitivity output metric. Total-order Sobol indices ($S_{T}$) were computed to quantify the proportion of output variance attributable to each parameter, including both main effects and higher-order interactions. The total-order index was selected as the primary sensitivity measure because it captures the complete contribution of each parameter to invasion variability within the multiscale pipeline.

To evaluate the stability of invasion metrics with respect to spatial discretisation, we performed a lattice-size sensitivity analysis. Agent-based simulations were repeated on square domains of L=200, L=300, and L=400 lattice units, while all biological parameters, intracellular rate mappings, and stochastic settings were kept identical. For each lattice size and scenario, n=30 stochastic replicates were generated, and median invasion radius ($radius_{95}$) and final occupied area were computed. Effect size relative to the L=200 reference domain was quantified using Cliff’s delta [50] to assess practical differences across discretisations.

## 5. Conclusions

We present a multiscale digital-twin framework integrating molecular docking, mechanistic ODE signalling, and agent-based modelling to study uPAR-driven prostate cancer invasion. MM-GBSA and induced-fit docking prioritised CAPE as a candidate uPA/uPAR modulator, while uPAR inhibition was implemented mechanistically at the signalling level rather than through direct energetic parametrisation. Coupling steady-state signalling outputs to spatial agent-based simulations demonstrated that uPAR inhibition leads to statistically significant reductions in tumour invasion and growth. In contrast, enhanced uPA signalling produced only modest, non-significant effects, highlighting the role of spatial and population-level constraints. This framework provides a scalable computational platform for translating molecular-scale prioritisation into quantitative predictions of tumour invasion dynamics.

## Figures and Tables

**Table 1:** Induced Fit Docking (IFD) scores for phytochemical ligands docked to uPAR.

Compound	Glide Score	Prime Energy	IFD Score
CAPE	−6.28	−269.77	−5269.8
Quercetin	−7.20	−270.15	−5259.0
Naringenin	−7.69	−270.17	−5249.6
Apigenin	−7.99	−269.75	−5235.1
Chrysin	−6.75	−268.41	−5233.2

**Table 2:** Median-based ranking of MM-GBSA binding free energies ($\Delta G_{\mathrm{bind}}$) for phytochemical–uPAR complexes.

Rank	Compound	*n*	Median	IQR	Mean	SD
1	CAPE	10	−35.05	9.81	−35.40	5.01
2	Quercetin	10	−27.66	4.69	−28.74	4.25
3	Chrysin	10	−24.30	8.85	−25.29	5.71
4	Apigenin	10	−23.05	6.04	−24.82	4.00
5	Naringenin	10	−22.32	5.98	−23.75	3.22

**Table 3:** Post hoc pairwise comparisons of MM-GBSA binding free energies using Dunn’s test with Benjamini–Hochberg correction, including non-parametric effect sizes.

Compound 1	Compound 2	Z	$p_{\mathrm{raw}}$	$q_{\mathrm{BH}}$	ΔMedian	Cliff’s δ	Magnitude
CAPE	Naringenin	−4.18	$2.91\times 10^{-5}$	$1.17\times 10^{-4}$	−12.73	−0.98	large
CAPE	Apigenin	−3.41	$6.61\times 10^{-4}$	$8.81\times 10^{-4}$	−12.01	−0.90	large
CAPE	Chrysin	−3.49	$4.83\times 10^{-4}$	$8.81\times 10^{-4}$	−10.75	−0.82	large
CAPE	Quercetin	−1.96	$4.96\times 10^{-2}$	$4.96\times 10^{-2}$	−7.40	−0.70	large

Interpretation: All pairwise comparisons remain statistically significant after Benjamini–Hochberg correction ($q_{\mathrm{BH}}<0.05$), with consistently large non-parametric effect sizes (Cliff’s δ from −0.70 to −0.98). Negative ΔMedian values indicate more favourable (more negative) $\Delta G_{\mathrm{bind}}$ for CAPE. ΔMedian denotes the difference in median $\Delta G_{\mathrm{bind}}$ values (Compound 1 minus Compound 2; kcal/mol). Cliff’s δ quantifies stochastic dominance of Compound 1 over Compound 2.

**Table 4:** Integrated multiscale results linking intracellular uPAR signalling (ODE) with emergent invasion phenotypes (ABM).

Scenario	ODE Steady-State	$r_{\mathrm{prol}}$ (Median ± IQR)	$r_{\mathrm{mot}}$ (Median ± IQR)	${\mathrm{radius}}_{95}$ (Median ± IQR)	Final Area (Median ± IQR)	Rank
${\mathrm{uPAR}}_{\mathrm{inhib}}$	Reduced signalling	∼0.035 ± IQR	∼0.059 ± IQR	**12.18** ± **IQR**	**408** ± **IQR**	1
${\mathrm{uPA}}_{\mathrm{high}}$	Enhanced signalling	∼0.036 ± IQR	∼0.061 ± IQR	12.44 ± IQR	401 ± IQR	2
Baseline	Physiological	∼0.036 ± IQR	∼0.061 ± IQR	12.53 ± IQR	415 ± IQR	3
**Kruskal–Wallis test (global effect)**						
$\chi_{{\mathrm{radius}}_{95}}^{2}$ p<0.05; $\chi_{\mathrm{area}}^{2}$ p<0.05						
**Post hoc Dunn test with Benjamini–Hochberg correction (vs. baseline)**						
${\mathrm{uPAR}}_{\mathrm{inhib}}$: **significant reduction in invasion** ($q_{\mathrm{BH}}<0.05$); ${\mathrm{uPA}}_{\mathrm{high}}$: trend toward increased spread						

Steady-state ODE outputs were computed from the final simulation window (24–72 h) and mapped onto agent-level phenotypes. ABM invasion endpoints were evaluated at the final simulation time and summarised using robust statistics (median and interquartile range). Scenario ranking is based on median spatial invasion (${radius}_{95}$), with lower values indicating reduced invasive potential. Bold values indicate statistically significant differences relative to baseline (Dunn’s test with Benjamini–Hochberg correction).

## Data Availability

The code and data used for this paper are available at Zenodo with the DOI: https://doi.org/10.5281/zenodo.18761670.

## Appendix A

### Appendix A.1. ODE System

Ligand–receptor binding and turnover

(A1) $$\frac{dL}{dt}=k_{L}^{\mathrm{prod}}-k_{\mathrm{on}}LR+k_{\mathrm{off}}C-k_{L}^{\deg}L$$

(A2) $$\frac{dR}{dt}=k_{R}^{\mathrm{prod}}-k_{\mathrm{on}}LR+k_{\mathrm{off}}C-k_{R}^{\mathrm{int}}R$$

(A3) $$\frac{dC}{dt}=k_{\mathrm{on}}LR-k_{\mathrm{off}}C-k_{C}^{\mathrm{int}}C$$

Here, $k_{on}$ and $k_{off}$ are the association and dissociation rates of uPA and uPAR, while $k_{R}^{int}$ and $k_{C}^{int}$ represent internalisation or removal of free receptor and ligand–receptor complexes, respectively.

FAK/Src activation by uPA-uPAR

(A4) $$\frac{dF}{dt}=k_{F}^{\mathrm{act}}C\left(F_{\mathrm{tot}}-F\right)-k_{F}^{\mathrm{deact}}F$$

AKT activation downstream of FAK/Src

(A5) $$\frac{dA}{dt}=k_{A}^{\mathrm{act}}F\left(A_{\mathrm{tot}}-A\right)-k_{A}^{\mathrm{deact}}A$$

ERK activation downstream of AKT (and/or parallel cascades)

(A6) $$\frac{dE}{dt}=k_{E}^{\mathrm{act}}A\left(E_{\mathrm{tot}}-E\right)-k_{E}^{\mathrm{deact}}E$$

NF−κB activation downstream of ERK

(A7) $$\frac{dN}{dt}=k_{N}^{\mathrm{act}}E\left(N_{\mathrm{tot}}-N\right)-k_{N}^{\mathrm{deact}}N$$

Implementation of uPAR inhibition

In simulations of uPAR blockade, the effective signalling through the uPA-uPAR complex can be reduced by the following:

- Scaling the available receptor *R*.
- Scaling the association rate as follows:

(A8) $$k_{\mathrm{on}}^{\mathrm{eff}}=\left(1-\eta \right)k_{\mathrm{on}},$$

where 0≤η≤1 is the fraction of uPAR blocked by inhibitor (η=0–no inhibition, η=1–full inhibition).

### Appendix A.2. List of Variables and Parameters

The parameters and their biological interpretation is set in Table A1.

**Table A1:** List of parameters with their biological interpretations used in the ODE system.

Parameter	Biological Meaning
$k_{L}^{\mathrm{prod}}$	uPA production/influx rate
$k_{L}^{\deg}$	uPA degradation/clearance rate
$k_{R}^{\mathrm{prod}}$	uPAR synthesis/recycling rate
$k_{R}^{\mathrm{int}}$	Internalisation/loss of free uPAR
$k_{C}^{\mathrm{int}}$	Internalisation/degradation of uPA–uPAR complexes
$k_{\mathrm{on}},\;k_{\mathrm{off}}$	Binding/unbinding rates of the uPA–uPAR interaction
$k_{F}^{\mathrm{act}},\;k_{F}^{\mathrm{deact}}$	Activation/deactivation rates of FAK/Src
$k_{A}^{\mathrm{act}},\;k_{A}^{\mathrm{deact}}$	Activation/deactivation rates of AKT
$k_{E}^{\mathrm{act}},\;k_{E}^{\mathrm{deact}}$	Activation/deactivation rates of ERK
$k_{N}^{\mathrm{act}},\;k_{N}^{\mathrm{deact}}$	Activation/deactivation rates of NF−κB
$F_{\mathrm{tot}},\;A_{\mathrm{tot}},\;E_{\mathrm{tot}},\;N_{\mathrm{tot}}$	Total cellular pools of FAK, AKT, ERK, and NF−κB

### Appendix A.3. MM-GBSA Results

**Table A2:** Raw MM-GBSA binding free energy components for phytochemical–uPAR complexes.

Compound	Replica	$\Delta G_{\mathrm{bind}}$	$\Delta G_{\mathrm{Coulomb}}$	$\Delta G_{\mathrm{Covalent}}$	$\Delta G_{\mathrm{Hbond}}$	$\Delta G_{\mathrm{Lipophilic}}$	$\Delta G_{\mathrm{vdW}}$	$\Delta G_{\mathrm{Solvation}}$
CAPE	R1	−37.39	−11.89	1.43	−1.14	−11.79	16.16	−28.86
CAPE	R2	−42.31	−14.92	2.48	−1.73	−13.37	18.53	−32.10
CAPE	R3	−30.94	−8.63	1.82	−0.60	−13.24	12.18	−20.03
CAPE	R4	−32.23	−9.14	1.39	−0.54	−12.24	13.28	−22.89
CAPE	R5	−32.71	−12.88	2.05	−1.33	−11.17	16.23	−24.29
CAPE	R6	−28.64	−9.53	1.83	−0.81	−10.22	15.07	−23.61
CAPE	R7	−37.50	−12.57	1.20	−1.02	−12.72	16.46	−27.21
CAPE	R8	−30.35	−10.66	1.82	−1.08	−9.62	14.49	−24.04
CAPE	R9	−41.13	−11.22	1.72	−0.70	−15.10	16.75	−30.01
CAPE	R10	−40.75	−13.09	1.69	−1.74	−12.90	19.37	−33.06
Quercetin	R1	−30.99	−17.28	2.19	−1.84	−4.66	17.24	−24.15
Quercetin	R2	−37.13	−22.66	2.96	−2.25	−5.91	21.53	−27.99
Quercetin	R3	−32.43	−15.37	2.11	−1.14	−5.10	18.74	−28.24
Quercetin	R4	−24.34	−11.72	1.54	−1.00	−4.32	13.43	−20.08
Quercetin	R5	−28.05	−14.81	1.84	−1.40	−4.95	16.00	−21.82
Quercetin	R6	−22.64	−10.98	1.59	−1.07	−3.88	10.93	−16.12
Quercetin	R7	−27.26	−13.61	1.80	−1.32	−5.13	17.07	−23.21
Quercetin	R8	−26.40	−16.21	1.59	−1.14	−4.41	17.28	−21.91
Quercetin	R9	−27.02	−14.36	1.63	−1.18	−4.27	16.75	−23.77
Quercetin	R10	−31.09	−16.76	2.05	−1.48	−4.90	18.43	−25.69
Naringenin	R1	−27.10	−13.61	1.01	−0.61	−4.69	17.00	−24.58
Naringenin	R2	−22.35	−9.98	1.01	−0.64	−4.32	11.56	−18.39
Naringenin	R3	−21.12	−9.53	1.14	−0.66	−3.82	11.42	−18.39
Naringenin	R4	−24.74	−12.01	0.87	−0.58	−4.23	14.09	−21.57
Naringenin	R5	−28.82	−14.16	1.32	−0.77	−4.79	15.84	−24.75
Naringenin	R6	−20.17	−10.07	1.06	−0.66	−3.50	12.75	−18.11
Naringenin	R7	−20.97	−8.94	0.83	−0.49	−3.80	11.48	−18.63
Naringenin	R8	−28.18	−14.58	1.53	−0.97	−4.94	17.57	−25.16
Naringenin	R9	−22.29	−9.43	0.75	−0.50	−3.91	12.49	−20.13
Naringenin	R10	−21.76	−10.39	0.67	−0.64	−4.00	12.25	−18.38
Apigenin	R1	−23.42	−8.16	1.22	−0.54	−4.67	11.55	−20.30
Apigenin	R2	−22.70	−8.21	1.14	−0.66	−4.95	11.74	−18.90
Apigenin	R3	−29.85	−17.91	1.30	−1.31	−6.18	21.27	−25.26
Apigenin	R4	−32.61	−13.08	0.76	−0.80	−6.78	10.87	−20.63
Apigenin	R5	−21.25	−8.93	0.95	−0.71	−4.28	10.68	−17.07
Apigenin	R6	−21.30	−8.41	1.28	−0.84	−4.70	12.15	−18.62
Apigenin	R7	−22.58	−7.63	1.43	−0.62	−4.99	10.19	−18.32
Apigenin	R8	−22.53	−10.25	0.98	−0.60	−4.44	12.87	−18.26
Apigenin	R9	−23.39	−7.55	0.96	−0.69	−5.12	10.81	−19.23
Apigenin	R10	−28.57	−11.97	0.97	−0.72	−5.16	15.90	−23.17
Chrysin	R1	−22.29	−5.68	0.63	−0.43	−4.06	9.15	−18.64
Chrysin	R2	−18.41	−5.78	0.70	−0.31	−2.99	8.36	−17.02
Chrysin	R3	−29.93	−6.81	0.60	−0.48	−6.13	11.10	−21.36
Chrysin	R4	−21.08	−5.81	0.49	−0.41	−4.23	8.25	−17.22
Chrysin	R5	−28.27	−6.37	0.56	−0.36	−6.04	9.87	−19.99
Chrysin	R6	−18.39	−7.06	0.51	−0.64	−2.95	8.66	−13.98
Chrysin	R7	−34.68	−10.79	0.49	−0.55	−7.06	13.60	−24.28
Chrysin	R8	−21.93	−3.57	0.47	−0.15	−5.09	6.64	−16.44
Chrysin	R9	−31.65	−13.31	1.19	−0.80	−5.95	17.14	−26.14
Chrysin	R10	−26.31	−9.15	1.00	−0.45	−4.46	13.41	−23.70

The raw MM-GBSA binding free energy components are reported for each phytochemical–uPAR complex. Five independent replicas (rR1–R10) were analysed per compound. All energies are reported in kcal/mol. These raw values were used to compute descriptive statistics, non-parametric comparisons, and compound prioritisation presented in the main text.

**Table A3:** Residue-level MM-GBSA energy decomposition for the CAPE–uPAR complex.

Residue	$\Delta G_{\mathrm{bind}}$	Coulomb	Solvation	vdW	H-Bond	Lipophilic
A:14 (LEU)	**−3.45**	−0.17	0.06	**−1.75**	0.00	**−1.59**
A:15 (ASN)	**−1.68**	−1.29	0.90	−0.78	−0.15	−0.20
A:43 (GLU)	**−1.52**	**−1.54**	1.43	−0.77	−0.20	−0.44
A:59 (ARG)	**−1.89**	−1.18	0.76	−0.86	−0.05	−0.42
A:92 (LEU)	**−2.76**	−0.27	0.51	**−1.80**	−0.01	**−1.19**
A:93 (GLN)	**−1.30**	−0.24	0.58	−1.04	−0.01	−0.43
Z:999 (UNK)	**−15.13**	**−6.54**	8.01	**−12.29**	−0.67	**−5.76**

Residue-level contributions were obtained from MM-GBSA energy decomposition. Bold values highlight residues with dominant contributions to binding ($|\Delta G_{\mathrm{bind}}|\;\geq 1.0$ kcal/mol or strong hydrophobic interactions). Residues with near-zero energetic contributions across all MM-GBSA components were excluded to improve readability and focus on functionally relevant interactions. All energies are reported in kcal/mol.

### Appendix A.5. Sensitivity Analysis

**Table A4:** Time-averaged effective proliferation and motility rates derived from the ODE model for each simulated scenario.

Scenario	η	*I* (μM)	${\bar{r}}_{\mathrm{prol}}$	${\bar{r}}_{\mathrm{mot}}$
Baseline	0	–	0.035894	0.062282
${\mathrm{uPA}}_{\mathrm{high}}$	0	–	0.035909	0.062362
${\mathrm{uPAR}}_{\mathrm{inhib},\mathrm{CAPE}0.1\;\mu M}$	0.0636	0.1	0.035894	0.062280
${\mathrm{uPAR}}_{\mathrm{inhib},\mathrm{CAPE}0.3\;\mu M}$	0.1615	0.3	0.035893	0.062274
${\mathrm{uPAR}}_{\mathrm{inhib},\mathrm{CAPE}1\;\mu M}$	0.3500	1	0.035889	0.062253
${\mathrm{uPAR}}_{\mathrm{inhib},\mathrm{CAPE}3\;\mu M}$	0.5250	3	0.035885	0.062235
${\mathrm{uPAR}}_{\mathrm{inhib},\mathrm{CAPE}10\;\mu M}$	0.6364	10	0.035882	0.062218

*η* denotes the occupancy-based inhibition parameter derived from docking-informed affinity mapping. ${\bar{r}}_{\mathrm{prol}}$ and ${\bar{r}}_{\mathrm{mot}}$ represent the time-averaged effective proliferation and motility rates used as inputs to the ABM layer.
